# Antifungal Activity, Structural Stability, and Immunomodulatory Effects on Human Immune Cells of Defensin from the Lentil *Lens culinaris*

**DOI:** 10.3390/membranes12090855

**Published:** 2022-08-31

**Authors:** Ekaterina I. Finkina, Ivan V. Bogdanov, Anastasia A. Ignatova, Marina D. Kanushkina, Ekaterina A. Egorova, Alexander D. Voropaev, Elena A. Stukacheva, Tatiana V. Ovchinnikova

**Affiliations:** 1 M.M. Shemyakin & Yu.A. Ovchinnikov Institute of Bioorganic Chemistry, The Russian Academy of Sciences, Miklukho-Maklaya St. 16/10, 117997 Moscow, Russia; 2 G.N. Gabrichevsky Research Institute for Epidemiology and Microbiology, Admiral Makarov St. 10, 125212 Moscow, Russia

**Keywords:** plant defensin, lentil *Lens culinaris* Lc-def, *Candida albicans*, candidiasis, antifungal activity, proteolytic stability, immunomodulatory effects, cytokines, multiplex xMAP assay

## Abstract

An increase in the frequency of mycoses and spreading of multidrug-resistant fungal pathogens necessitates the search for new antifungal agents. Earlier, we isolated the novel defensin from lentil *Lens* *culinaris* seeds, designated as Lc-def, which inhibited the growth of phytopathogenic fungi. Here, we studied an antifungal activity of Lc-def against human pathogenic *Candida* species, structural stability of the defensin, and its immunomodulatory effects that may help to prevent fungal infection. We showed that Lc-def caused 50% growth inhibition of clinical isolates of *Candida albicans*, *C. krusei*, and *C. glabrata* at concentrations of 25–50 μM, but was not toxic to different human cells. The lentil defensin was resistant to proteolysis by *C. albicans* and was not cleaved during simulated gastroduodenal digestion. By using the multiplex xMAP assay, we showed for the first time for plant defensins that Lc-def increased the production of such essential for immunity to candidiasis pro-inflammatory cytokines as IL-12 and IL-17 at the concentration of 2 μM. Thus, we hypothesized that the lentil Lc-def and plant defensins in general may be effective in suppressing of mucocutaneous candidiasis due to their antifungal activity, high structural stability, and ability to activate a protective immune response.

## 1. Introduction

Currently, an increase in the frequency of fungal infections has become a severe problem. More than 1.5 million people die from fungal diseases every year [1]. The people most susceptible to mycoses are those with an immunodeficiency state, which may be the result of HIV infection, as well as immunosuppressive therapy for malignant tumors, autoimmune diseases, and organ transplantation. The latest data also show that COVID-19 is often accompanied by opportunistic fungal infections [2]. 

*Candida* spp. are the most common causative microorganisms of fungal opportunistic infections. *Candida* infections vary in severity from superficial mucosal candidiasis to life-threatening blood stream infection candidemia and invasive candidiasis, that affects multiple organs and is characterized by high mortality [3,4]. *Candida albicans* is the most common cause of candidiasis, but other *Candida* species, including *C. glabrata* and *C. krusei*, contribute to the spreading of this fungal infection. Immune sensing of *Candida* leads to activation of strong immune response, production of pro-inflammatory cytokines, in particular IL-12, IL-17, and IFN-γ, recruitment of immune cells, and synthesis of protective antimicrobial peptides (AMPs) such as β-defensins, cathelicidin LL-37, and histatin [5]. It is known that the decrease of IL-17 levels taking place in HIV-infected patients and patients with genetic defects of IL-17 immunity, predisposes to the development of mucocutaneous candidiasis [6,7]. On the other hand, *Candida* has a number of immune evasion mechanisms, including the production of a variety of proteolytic enzymes, which effectively cleave AMPs and other protective immune molecules [4]. 

The list of conventional antimycotics is quite limited. Furthermore, antifungal drugs are characterized by high toxicity [8]. In addition, spreading of multidrug-resistant fungal strains takes place. Resistance to azole derivatives and echinocandins in fungi of the *Candida* genera, especially *Candida glabrata*, is common in clinical practice [9]. All this together makes the search for new antifungal agents a high priority task. 

Defensins constitute a conservative class of AMPs, found in animals, plants, fungi, and bacteria. Despite a low level of sequence homology, most defensins have a somewhat similar compact spatial structure, stabilized by disulfide bonds [10]. Unlike animal defensins, showing a broad spectrum of antimicrobial action, plant defensins act mostly against fungi, which are the main plant pathogens [11]. Plant defensins are characterized by a variety of mechanisms of antifungal action, based on interaction with different lipid components of the membranes specific for fungi, for example, glucosylceramides. Therefore, they are not toxic, as a rule, towards human cells [12]. Moreover, some plant defensin, for example the pea Psd1 [13], radish RsAFP1 and RsAFP2 [14], and PvD1 from the common bean *Phaseolus vulgaris* [15], possess a pronounced antifungal activity also towards human pathogenic *Candida* species due to the similarity of their cell membrane structure. Therefore, defensins are considered promising objects to create possible prototypes of new antifungal agents.

It is shown that mammal AMPs not only effectively inhibit the growth of pathogenic microorganisms, but also affect host immune system particularly, but not exclusively under infection. For instance, human defensins stimulate migration of immune cells, promote the release of pro-inflammatory cytokines, and recruit antigen-presenting cells, as well as facilitate the decrease in the inflammatory response upon bacterial infection [16,17]. However, much less is known about an ability of foreign AMPs to influence the human immune cells. In the case of plant defensins, immunomodulatory effects of only two peptides have been studied to date. It was shown that defensin from *Capsicum chinense* modulates the innate immune response of bovine mammary epithelial cells infected with *Staphylococcus* aureus by inducing the production of both pro-inflammatory cytokines TNF-α and IL-1β and anti-inflammatory IL-10 [16]. The designed analogue of plant defensins EgK5 suppresses antigen-triggered proliferation of effector memory T cells, a subset enriched among pathogenic autoreactive T cells in autoimmune disease [18]. Certainly, more experimental data are needed to clarify the potential of plant defensins as AMPs with immunomodulatory activity.

Earlier, we discovered and characterized the novel defensin from the lentil *Lens culinaris* seeds, designated as Lc-def, which possesses an antifungal activity against a variety of phytopathogenic fungi [19,20]. The main goal of this study was to investigate the antimicrobial activity of Lc-def against human pathogenic fungi as well as its possible immunomodulatory effects reducing the risk of infection. We studied an ability of lentil defensin to inhibit the growth of clinical isolates of various *Candida* species and examined the sensitivity of Lc-def to heating, pH changes, and cleavage by *C. albicans* proteolytic enzymes as well as by proteases from human gut. Furthermore, for the first time for plant defensins, we investigated the ability of Lc-def to induce cytokine response by different human immune cells by using the multiplex xMAP assay.

## 2. Materials and Methods

### 2.1. Materials

Digestive enzymes (porcine pepsin and trypsin, bovine α-chymotrypsin), model protein substrate bovine α-casein, and secondary anti-rabbit IgG polyclonal goat antibodies conjugated to HRP and 3,3′,5,5′-tetramethylbenzidine (TMB) substrates were purchased from Sigma-Aldrich (St. Louis, MO, USA). Synthetic lysophospholipids (lyso-palmitoyl phosphatidylcholine (LPPC), lyso-palmitoyl phosphatidylglycerol (LPPG)) were purchased from Avanti Polar Lipids (Alabaster, AL, USA).

The recombinant Lc-def (UniProt:B3F051) was overexpressed in *Escherichia coli* and purified as described previously [20]. Repeated RP-HPLC (reversed-phase high performance liquid chromatography) on a Luna C_18_ column (5 μm, 250 × 4.6 mm; Phenomenex, Torrance, CA, USA) with a linear gradient of acetonitrile in 0.1% TFA was used for additional purification of recombinant Lc-def samples for cytokines response assays. The recombinant Lc-LTP2 was obtained as described [21]. Melittin and LL-37 (>98% pure for both peptides) were synthesized using a standard solid-phase method in M.M. Shemyakin and Yu.A. Ovchinnikov Institute of Bioorganic Chemistry of the Russian Academy of Sciences (Moscow, Russia). 

The clinical isolates of *Candida albicans* (v47a3), *C. krusei* (225/2) and *C. glabrata* (252/2) were collected from patients with human immunodeficiency virus (HIV) infection and provided by G.N. Gabrichevsky Research Institute for Epidemiology and Microbiology (Moscow, Russia). Strains stocks were kept at −70 ℃ in 10% nonfat milk with 10% glycerol.

### 2.2. Heterologous Expression and Purification of Recombinant Psd1

DNA fragment encoding pea *Pisum sativum* defensin, designated as Psd1 (UNIPROT P81929), was obtained by de novo synthesis using PCR with overlapping primers (Supporting Materials, Table S1). The resulting fragment was inserted into the expression plasmid vector pET-His8-TrxL pre-treated with BamHI by ligase-free cloning method. The *E. coli* BL-21 (DE3) cells transformed with pET-His8-TrxL-Psd1 were grown in LB (Luria–Bertani) liquid medium containing ampicillin and glucose. Recombinant Psd1 was purified as described for lentil Lc-def [20]. Homogeneity and the identity of the recombinant peptide sample was confirmed by MALDI mass spectrometry and CD spectroscopy (Supporting Materials, Figures S1 and Table S2). 

### 2.3. Mass Spectrometry

All mass spectrometry measurements were performed by using Ultraflex III MALDI-TOF mass spectrometer (Bruker, Bremen, Germany) equipped with a UV Nd:YAG laser (355 nm) and LIFT MS/MS unit. The molecular weights of the recombinant Lc-def and digestion products were determined in a linear and reflector mode.

### 2.4. CD Spectroscopy 

Circular dichroism spectra were recorded at different temperatures using a J-810 spectropolarimeter (Jasco, Hachioji, Tokyo, Japan) in a 0.1 cm path length quartz cell (Hellma GmbH and Co. KG, Mullheim, Germany) in the 190–250 nm range 0.2 using solutions of Lc-def in 10 mM phosphate buffer, pH 7.4, or 150 mM sodium chloride, pH 2.5, simulated gastric fluid, at a concentration of 30 µM. Lysolipids were added at final concentration of 1 mM.

### 2.5. Antifungal Assay

Antifungal susceptibility tests were performed by the microdilution method using 96-well microplates. Yeast cells in stock were inoculated onto modified YPD (yeast extract 5 g/L, peptone 10 g/L, glucose 10 g/L) agar plates, and incubated for 24 h at 37 ℃. After replating, cells were cultured in modified YPD broth at 37 ℃ to optical density 1.0 at 540 nm. Further, yeast cells in modified YPD broth at concentration of 5 × 10^4^ cells/mL were mixed with equal volumes of serial two-fold dilutions of the peptide in water which started at the highest peptide concentration of 200 µM. Final peptide concentrations in the wells were 100, 50, 25, 12.5, 6.25, 3.125, and 1.56 μM. Controls without defensin were also tested. Plates were incubated at 30 ℃ during 24 h. Yeast growth was estimated using inverted microscope as well as by measuring the optical density at 540 nm. Percentage of growth inhibition was defined as: growth inhibition (%) = ((A_control_ − A_sample_)/A_control_) × 100%. All of the experiments were performed twice in triplicate. The *t*-test was performed to study the statistical difference (*p*-value) between the means of controls and samples with different peptide concentrations. The experiment with lentil *Lens culinaris* lipid transfer protein Lc-LTP2 was carried out for comparison.

### 2.6. Sensitivity to Proteolytic Enzymes of Candida albicans

For study of Lc-def sensitivity to the clinical isolate of *C. albicans*, lysates of yeast cells in different concentration and pH were used basically as previously described for rhesus macaque θ-defensin 1 [22]. Cells were inoculated onto modified YPD medium and grown at 37 ℃ for 48 h before reaching the stationary phase of growth. Cells were harvested by centrifugation, washed two times in sterile phosphate buffered saline (PBS), pH 7.2, or sterile 40 mM sodium acetate, pH 4.0, and resuspended in the corresponding buffer to final concentrations of 1 × 10^9^ cells/mL or 1 × 10^3^ cells/mL. Two freeze-thaw cycles in liquid nitrogen were used for cell disruption [23], and supernatants were collected following centrifugation. Supernatants (12.5 μL) were mixed with water solutions (37.5 μL) of Lc-def, α-casein, Psd1, or LL-37 in concentration of 0.67 mg/mL and incubated at 37 ℃ for 72 or 24 h in the case of high or low cell concentration, respectively. To study the possible influence of Lc-def and Psd1 on the degradation process, supernatants (12.5 μL) were mixed with both α-casein or LL-37 in concentration of 1 mg/mL (25 μL) and Lc-def or Psd1 in concentration of 2 mg/mL (12.5 μL). As negative controls, α-casein and peptides were mixed with buffers as well as supernatants were mixed with water without the proteins. The samples were mixed with sample buffer without β-mercaptoethanol (BME), heated at 100 ℃ for 2 min, and analyzed by sodium dodecyl sulfate polyacrylamide gel electrophoresis (SDS-PAGE). Some independent experiments were performed in each case.

### 2.7. Sensitivity to Digestive Enzymes

Sensitivity of Lc-def to digestive enzymes was examined by simulation of its gastrointestinal digestion in vitro. Gastric digestion was performed for 2 h, using 50 ng of pepsin per 1 μg of preheated at 100 °C for 30 min and intact Lc-def in 0.1 M HCl, pH 2.0. For duodenal digestion, the pH of the mixture resulting from gastric digestion was adjusted to 8.0 by addition of ammonium bicarbonate. The obtained mixture was incubated for 24 h at 37 °C with 2.5 ng of trypsin and 10 ng of α-chymotrypsin per 1 μg of the defensin. The degree of proteolysis was monitored by SDS-PAGE and RP-HPLC. Gel analysis was performed using Gel Doc XR^+^ imaging system (Bio-Rad, Hercules, CA, USA) and Image Lab Software v.6.1.0 (Bio-Rad, Hercules, CA, USA). Some independent experiments were performed in each case. The experiment with α-casein was carried out for comparison.

### 2.8. Antibodies Preparation and Immunoblotting

Polyclonal anti-Lc-def antibodies were prepared by immunization of two rabbits. At the first stage, the rabbits were subcutaneously administered by recombinant Lc-def (150 μg/rabbit) with complete Freund’s adjuvant. After that, a half dose of the antigen with incomplete Freund’s adjuvant and, finally, the recombinant peptide in PBS were used. Sera samples obtained from the same rabbits prior to immunization were used as a negative control.

Monitoring of the anti-Lc-def IgG titer and study of cross-reactivity of antibodies obtained was carried out using ELISA. Recombinant lentil Lc-def or pea Psd1 were immobilized in PBS. After plate blocking with 2% BSA in PBS, immune and pre-immune sera samples were added for overnight incubation at 4 ℃. After a washing step with PBS-T, secondary anti-rabbit IgG polyclonal goat antibodies conjugated to HRP (in 1:50,000 dilution) were added. After 2 h incubation at 37 ℃ and washing step with PBS-T, immune complexes were visualized using TMB as a chromogenic substrate (Supporting Materials, Figure S2A). 

Immunoblotting was performed using rabbit anti-Lc-def antibodies in a 0.5% nonfat dry milk solution in PBS in 1:25 dilution. Following SDS-PAGE, the proteins were electrotransferred to a nitrocellulose membrane (0.2 mkm, Millipore, Bedford, MA, USA) in a buffer containing 20% of methanol and 0.1% of SDS. After a washing step in PBS, the membrane was blocked for one hour using 2% milk in PBS. After the washing step in PBS-T, the membrane incubation with anti-Lc-def antibodies during overnight at 4 ℃ was carried out. After the washing step in PBS-T, the membrane was incubated with secondary antibodies in 1% milk in PBS (in 1:10,000 dilution) during 2 h at 37 ℃. Detection was carried out using TMB solution for membranes (Supporting Materials, Figure S2B,C).

### 2.9. Human Cell Cultures

Colorectal adenocarcinoma Caco-2 cell line (ATCC HTB-37) was cultured in complete DMEM/F12 (1:1) medium containing 10% fetal bovine serum (FBS, Gibco) and 1X antibiotic-antimycotic solution (1XAAS, Invitrogen, Waltham, MA, USA). The acute monocytic leukemia THP-1 line (ATCC TIB-202) was cultured in complete RPMI-1640 medium, containing 10% FBS, 1XAAS, and 20 mM BME. THP-1 cells were differentiated into pro-inflammatory macrophages (Mφ1) with phorbol 12-myristate 13-acetate (Sigma-Aldrich) for 24 h and subsequently polarized with lipopolysaccharide from *E. coli* (Sigma-Aldrich) and rhIFN-γ (Sigma-Aldrich) for another 48 h according to previously reported protocol [24]. All cells were cultured in a humidified CO_2_ incubator (5% CO_2_, 37 °C). Primary peripheral blood mononuclear cells (PBMCs) collected from a healthy donor were purchased from American Type Culture Collection (ATCC PCS-800-011). CD4^+^ T-helper and CD8^+^ T cytotoxic cells were isolated from PBMCs by magnetic separation with mouse anti-human CD4-PE (clone 13B8.2, Beckman Coulter Diagnostics, Pasadena, CA, USA) and CD8-PE (clone REA734, Miltenyi Biotec, Bergisch Gladbach, Germany), Anti-PE MicroBeads (Miltenyi Biotec), and LS Separation Columns (Miltenyi Biotec). Isolated CD4^+^ and CD8^+^ cells were expanded separately with Dynabeads™ Human T-Expander CD3/CD28 (Gibco, Waltham, MA, USA) in RPMI-1640 supplemented with 10% FBS, 1XAAS and 100 U/mL rh-IL-2 (Gibco). Regulatory CD4^+^ FoxP3^+^ T cells (Tregs) were isolated from PBMCs by magnetic separation with CD4^+^CD25^+^ Regulatory T Cell Isolation Kit, human (Miltenyi Biotec). Primary CD14^+^ blood monocytes were isolated from PBMCs by adherence according to standard technique [25]. Monocyte-derived mature dendritic cells (moDCs) were obtained from CD14^+^ blood monocytes according to the previously reported protocol [26]. Briefly, blood monocytes were incubated in complete RPMI-1640 medium, containing 10% FBS, 1XAAS, 800 U/mL rhGM-CSF (Sci-Store, Russia, Moscow) and 500 U/mL rhIL-4 (Sci-Store) for 3 days. Fresh complete RPMI-1640 medium with rhGM-CSF and rhIL-4 was added to the culture on day 3. On day 5, fresh complete RPMI-1640 containing 100 U/mL rhTNF-α (Sigma) as well as 800 U/mL rhGM-CSF and 500 U/mL rhIL-4 was added and the culture was incubated for another 4 days until full maturation.

### 2.10. Cytokines/Chemokines/Growth Factors Production by Cell Cultures

Caco-2 cells were seeded into the wells of a 48-well plate, coated with 0.1% bovine collagen (Sigma-Aldrich) for 24 h, 3 weeks before the experiment with complete DMEM/F12 (1:1) medium renewal every 2–3 days. Monocytes, moDCs, CD4^+^ T-helper and CD8^+^ T cytotoxic cells, Mφ1, and Regulatory CD4^+^ FoxP3^+^ T cells were seeded into the wells of 48-well plates in the complete RPMI-1640 medium supplemented with 10% Human AB serum (HABS, Capricorn Scientific, Ebsdorfergrund, Germany) and 1XAAS 24 h prior to the experiment; medium in Caco-2-containing wells was also replaced with the same medium. After 24 h, half of the medium in each well was replaced by fresh complete RPMI-1640 medium with 10% HABS, supplemented with 4 µM of Lc-def for the sample wells or fresh medium alone for the control wells. Cell cultures were kept in CO_2_-incubator (5% CO_2_, 37 °C) for another 24 h. Culture supernatants were collected 24 h later and stored at −70 °C degrees less than one month prior to the analytes assessment.

### 2.11. Cytokine Response by Different Human Cell Cultures to Lentil Defensin

The following 48 analytes were measured at a protein level by multiplex xMAP technology using the MILLIPLEX Human Cytokine/Chemokine/Growth Factor Panel A kit (HCYTA-60K-PX48, Merck, Darmstadt, Germany): sCD40L, EGF, Eotaxin-1/CCL11, FGF-2/FGF-basic, Flt-3 ligand, Fractalkine/CX3CL1, G-CSF, GM-CSF, GROα, IFNα2, IFN-γ, IL-1α, IL-1β, IL-1RA, IL-2, IL-3, IL-4, IL-5, IL-6, IL-7, IL-8/CXCL8, IL-9, IL-10, IL-12(p40), IL-12(p70), IL-13, IL-15, IL-17A/CTLA8, IL-17E/IL-25, IL-17F, IL-18, IL-22, IL-27, IP-10/CXCL10, MCP-1/CCL2, MCP-3/CCL7, M-CSF, MDC/CCL22, MIG/CXCL9, MIP-1α/CCL3, MIP-1β/CCL4, PDGF-AA, PDGF-AB/BB, RANTES/CCL5, TGF-α, TNF-α, TNF-β, and VEGF-A. Multiplex-based assay read-out was performed using MAGPIX system (Merck) with the xPONENT 4.2 software (Merck) in accordance with the manufacturer’s instruction with overnight incubation of the samples with primary antibodies. Final analysis was carried out with the MILLIPLEX Analyst v5.1 software (Merck). Measurements were performed twice for each sample. Release of the analytes in control and experimental samples was compared with unpaired two-sample *t*-test using GraphPad Prism v.8.0.1 (GraphPad Software, Inc, San Diego, CA, USA). The *p* values ≤ 0.05 were considered significant.

### 2.12. Hemolytic and Cytotoxicity Assays

Hemolytic activity of Lc-def was studied using 96-well microplates. Fresh human red blood cells (hRBC) were washed three times with ice-cold PBS, pH 7.2. Serial two-fold dilutions of Lc-def and membrane-active peptide melittin from the venom of honeybees as positive control (final concentrations from 0.1 to 100 μM) were then added to hRBC in PBS (final concentration of 4% (*v*/*v*)). The suspension was incubated for 1.5 h at 37 °C. After, intact hRBC sedimentation aliquots of the supernatants containing released hemoglobin were transferred to 96-well microplates, and the absorbance at 405 nm was measured. hRBC in PBS or 0.1% Triton X-100 were employed as negative (control0) and positive (control100) controls, respectively. The percentage of hemolysis was calculated as: hemolysis(%) = ((A_sample_ − A_control0_)/(A_control100_ − A_control0_)) × 100%. Experiment was carried out twice in triplicate using the same blood.

The cytotoxic properties of Lc-def and melittin as a control were investigated also by resazurin-based cell cytotoxicity assay. PBMCs and THP-1 cells were seeded in 96-well plates at 2 × 10^6^ and 1 × 10^6^ cells per well, respectively, in RPMI-1640 supplemented with 10% FBS, and kept in CO_2_-incubator. After 24 h serial two-fold dilutions of compounds in culture medium were then added to final concentrations from 0.2 to 50 μM. After 24 h of incubation with the peptides, resazurin (Sigma) was added at a final concentration of 0.7 mM, and the plates were incubated overnight (16 h). Fluorescent resorufin was registered using 535/595 filter at PlateReader AF2200 (Eppendorf, Hamburg, Germany). Untreated corresponding cells were used as negative controls. The cell viability was calculated as: cell viability (%) = (F_sample_/F_control_) × 100%. The experiment was carried out twice in triplicate.

### 2.13. Statistical Data Analysis

Absolute values of the analytes in cell culture supernatants were normalized using a logarithmic transformation by LN function [27] in Microsoft Excel. LN-transformed values were used for comparing the analyte levels in control and experimental samples by unpaired two-sample *t*-test. The normality of distribution of the absorbances in wells with serial dilutions of Lc-def in antimicrobial assay was assessed using Shapiro–Wilk (*W*-test) and Kolmogorov–Smirnov tests. All data samples passed both normality tests with assigned α = 0.05. The absorbances in wells with serial dilutions of Lc-def in antimicrobial assay for all the three *Candida* species were compared by two-way ANOVA. Variation of the absorbances values through different concentrations of Lc-def was shown to be significant (*p* < 0.0001). The absorbances in wells, containing 1.56 µM Lc-def, were also compared with ones, containing 50 and 100 µM Lc-def, by unpaired two-sample *t*-test. Differences at *p* < 0.05 level were considered in the manuscript as statistically significant. All statistical data analysis was performed with PraphPad Prism 8.1.1 analytic package (Graphpad Software Inc., La Jolla, CA, USA).

## 3. Results

### 3.1. Effects of Different pH and Temperature on Stability of the Lc-def Structure

**Figure 1:**
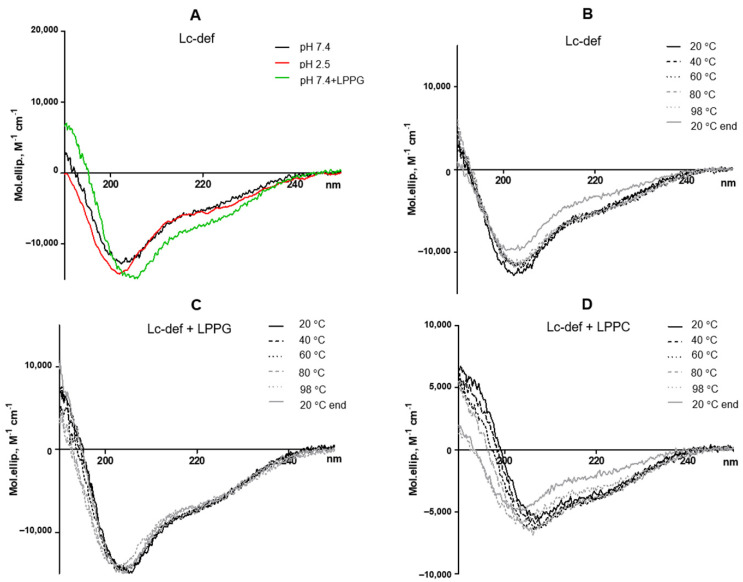
CD spectra of lentil *Lens culinaris* defensin at 20 °C at different pH without or in the presence of LPPG (**A**). Effects of heating up to 98.5 °C and subsequent cooling down to 20 °C on the secondary structure of Lc-def without (**B**) or in the presence of lysolipids LPPG (**C**) and LPPC (**D**) at final concentrations of 1 mM.

Stability of the Lc-def structure was examined by CD spectroscopy. The far-UV CD spectrum of Lc-def in 10 mM phosphate buffer, pH 7.4, showed a combination of α- and β-secondary structures with a positive maximum at 190 nm and two negative extremes at 206 and 222 nm. The presence in the solution of lysolipid LPPG as well as LPPC increased the percentage of α-helices in the Lc-def structure. The CD spectrum of Lc-def at pH 2.5 were partially the same, but revealed a decrease in the α-helix content (Figure 1A, Table 1). The spectrum of Psd1 at pH 7.4 was about the same as in the case of Lc-def (Table 1). 

The heating up to 98 °C led to a partial denaturation of the Lc-def structure at pH 7.4, and subsequent cooling down to 20 °C did not lead to a recovery of the native conformation. The presence of the negatively charged LPPG, by contrast with the neutrally charged LPPC, at the concentration of 1 mM at pH 7.4 prevented defensin thermal denaturation (Figure 1B–D, Table 1). 

### 3.2. Antifungal Activity of Lc-def

**Figure 2:**
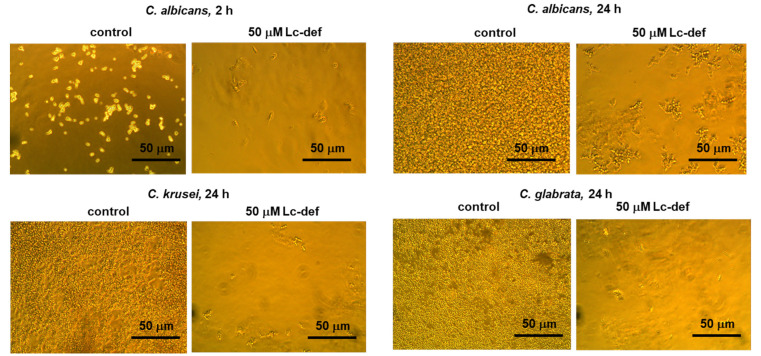
Effects of Lc-def on the growth of the clinical isolates of *C. albicans*, *C. krusei*, and *C. glabrata* (×400 magnification).

**Figure 3:**
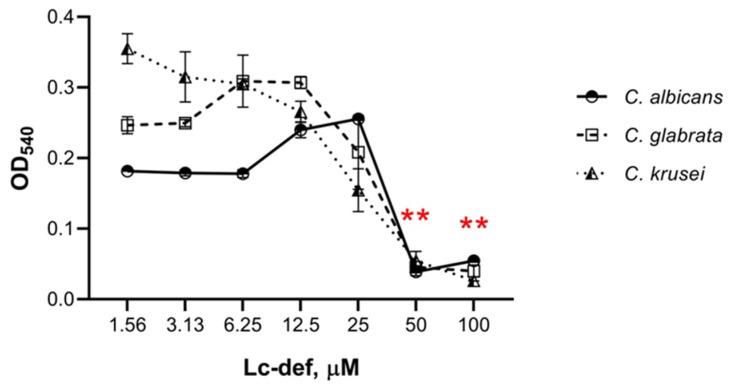
Antifungal activity of Lc-def at different concentrations (100, 50, 25, 12.5, 6.25, 3.125, and 1.56 μM) against the clinical isolates of *C. albicans*, *C. krusei*, and *C. glabrata* after incubation during 24 h. Error bars represent a standard deviation between two replications. A significant difference (**, *p* < 0.01) was observed for all the three *Candida* species for Ls-def at concentrations of 50 and 100 μM.

Lc-def inhibited the growth of all the three clinical isolates of *C. albicans*, *C. krusei*, and *C. glabrata* (Figure 2 and Figure 3). The effect of growth inhibition was noticeable after 2 h of incubation of yeast cells with the peptide (Figure 2). In control wells without peptide, an increase in cell concentration was observed under the microscope. In the presence of defensin at concentrations of 25–100 µM, cell growth was slower. Lc-def caused approximately 80% or 90% inhibition of the growth of *C. albicans* or *C. krusei* and *C. glabrata,* respectively, at the concentration of 50 μM. 100% Inhibition was not achieved even at 100 μM concentration of Lc-def. *C. krusei* was the most sensitive to Lc-def, and 50% growth inhibition was observed at the peptide concentration of 25 μM (Figure 3). Not only slowing down of cell growth, but also yeast cell disruption was observed at Lc-def concentrations of 50 and 100 μM under the microscope (Figure 2). In contrast, lentil lipid transfer protein Lc-LTP2, also showing an activity against phytopathogenic fungi [21], was not active against *Candida* species. Surprisingly, a growth activation was observed at the protein concentrations of 25–100 μM (Supporting Materials, Figure S3A). The optical density in these wells was significantly higher than in the control wells without Lc-LTP2. Possibly, the same effect of a protective activation of yeast growth was registered at 6.25–25 μM concentrations of Lc-def.

### 3.3. Hemolytic and Cytotoxicity Assays of Lc-def 

Lc-def did not cause hemolysis of erythrocytes even at the concentration of 100 μM. At the same time, 50% hemolysis was observed for the membranotropic peptide melittin at the concentration of 1.6 μM (Supporting Materials, Figure S3B). Lc-def was not toxic to PBMCs. Cell viability of 100% was observed even at the peptide concentration of 50 μM. At the same time, the death of about 50% of cells was observed for melittin at the concentration of 6.25 μM (data not shown).

### 3.4. Resistance of Lc-def to Proteolysis

**Figure 4:**
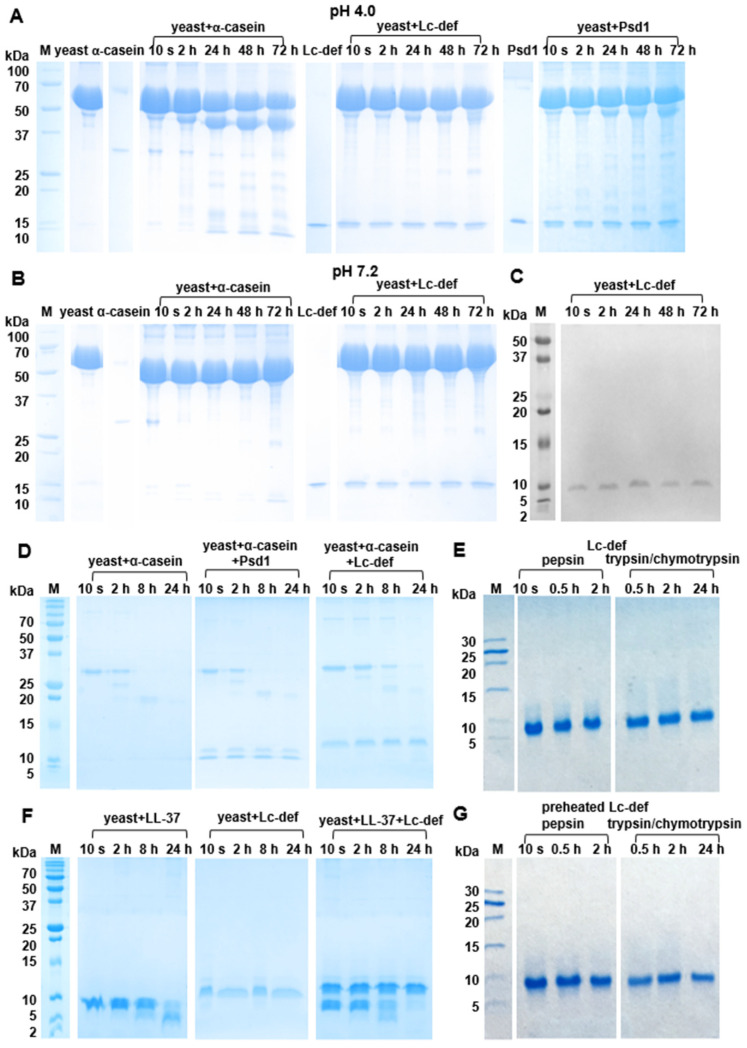
Resistance of Lc-def to proteolysis. (**A**,**B**) Results of α-casein, Lc-def, and Psd1 incubation with yeast (10^9^ cell/mL) in buffer solutions (pH 4.0 or 7.2) during 10 s and 2, 24, 48, and 72 h as detected by SDS-PAGE (15%T, 3%C in the separating gel). (**C**) Immunoblotting of aliquots obtained after Lc-def incubation with yeast (10^9^ cells/mL) in the buffer solution with pH 7.2 using polyclonal rabbit anti-Lc-def IgG. (**D**,**F**) Results of α-casein and LL-37 incubation with yeast (10^3^ cells/mL) in the buffer solution with pH 7.2 during 10 s, and 2, 8, and 24 h in the presence of or without Lc-def/Psd1 as detected by SDS-PAGE (15%T, 3%C or 18%T, 3%C in separating gel in the case of (**D**) or (**F**), respectively). (**E**,**G**) The results of a subsequent digestion of the intact or preheated Lc-def by pepsin (during 10 s, and 0.5 and 2 h) or by the mixture of trypsin/α-chymotrypsin (during 0.5, 2. and 24 h) as detected by SDS-PAGE (18%T, 3%C in the separating gel).

At first, a sensitivity of Lc-def to proteolysis by *C. albicans* was studied using the yeast sample with a high concentration (10^9^ cells/mL). In electropherograms of both samples with pH of 4.0 or 7.2, the major protein of *C. albicans* with the molecular mass of approximately 60 kDa was observed. α-Casein used as a model protein was highly susceptible to proteolysis and was degraded completely during 2 h in the case of pH 7.2 (Figure 4B). On the other hand, hydrolysis of α-casein was slow at pH 4.0 possibly due to the degradation of the major protein of *C. albicans* (Figure 4A). Lc-def as well as Psd1 were stable to proteolysis by yeast in both samples with pH 4.0 or 7.2 for 72 h (Figure 4A,B). Densitometric gel analysis as well as immunoblotting with rabbit anti-Lc-def antibodies confirmed the absence of the Lc-def degradation (Figure 4C). Immunoblotting was not performed in the case of Psd1 due to the low cross-reactivity of the rabbit anti-Lc-def IgG (Supporting Materials, Figure S4). It is worth noting that decrease in degradation of the major protein of *C. albicans* was observed in the presence of Lc-def and to a lesser extent in the presence of Psd1.

At the next stage, the yeast sample with a low cell concentration (10^3^ cells/mL), pH 7.2, was used to study an influence of Lc-def on degradation of α-casein as a model protein and the human antimicrobial peptide LL-37 (MW 4.5 kDa). There were no visible protein bands on the electropherogram of this yeast sample. α-Casein was degraded effectively under these conditions too and the band corresponding to the protein was absent in the electropherogram after 8 h. Lc-def but not Psd1 decreased the rate of α-casein degradation. The band corresponding to the protein was observed in the electropherogram even after incubation of α-casein in the presence of Lc-def during 24 h (Figure 4D). Hydrolysis of LL-37 by the yeast sample with a low cell concentration took place. According to the densitometric gel analysis, only about 30% of the intact peptide was present in the hydrolysate after 24 h. Surprisingly, Lc-def did not decrease LL-37 degradation (Figure 4F).

Further, a sensitivity of Lc-def to proteolytic enzymes of human gut was also examined. For that purpose, a consequent cleavage of Lc-def by pepsin and by the mixture of trypsin/α-chymotrypsin mimicking gastrointestinal digestion in vitro was performed. The lentil defensin showed a remarkable stability toward gastrointestinal digestion (Figure 4E). α-Casein, which was taken as a control, almost completely degraded by pepsin in the first 5 min under these conditions (data not shown). At the same time, preheating of the lentil defensin increased the sensitivity of Lc-def to proteolysis, possibly due to a partial peptide denaturation as it was shown by CD spectroscopy (Figure 4G). Densitometric gel analysis revealed approximately 30% degradation of the preheated Lc-def. This was also confirmed by RP-HPLC analysis of Lc-def digests (Supporting Materials, Figure S4A). The only peak in the chromatograms at 33 min, corresponding to the intact defensin, was detected in hydrolysates of the intact Lc-def by mass spectrometry analysis. At the same time, two additional peaks at 28 and 31 min containing proteolytic products of Lc-def were detected in hydrolysates of the preheated Lc-def (Supporting Materials, Figure S4B). 

### 3.5. Immunomodulatory Activity of Lc-def

**Figure 5:**
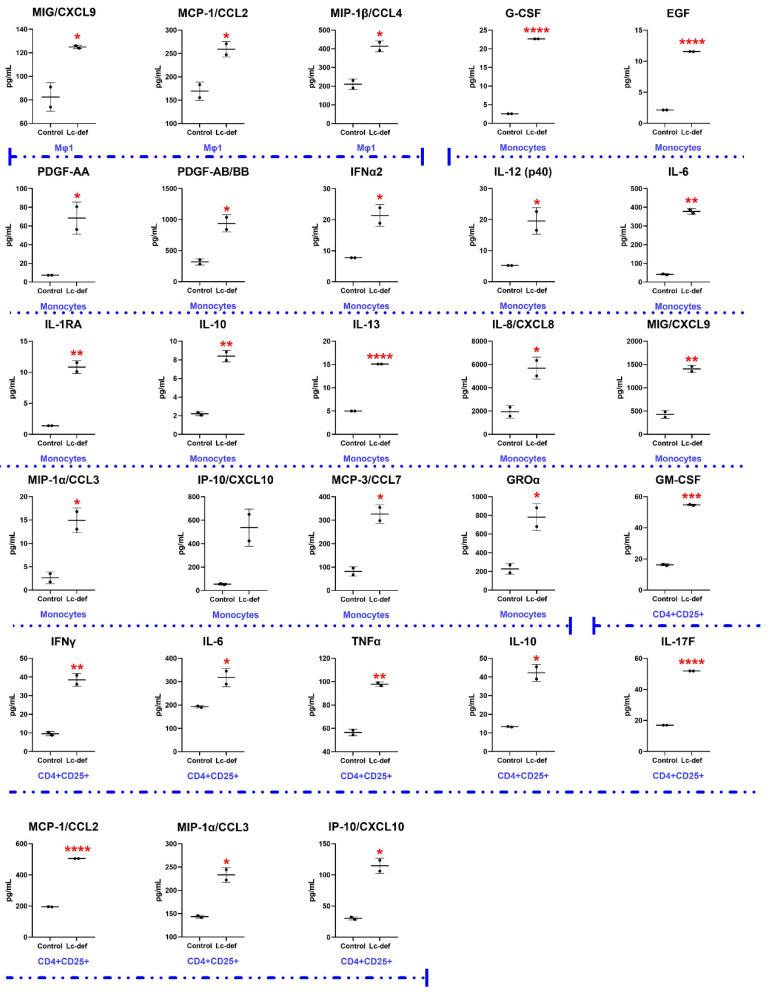
Profiles of cytokines and chemokines production by different cell cultures in vitro in response to Lc-def after incubation with 2 µM of the peptide. Error bars represent a standard deviation (±SD) between two replications. Significance levels are: * *p* < 0.05, ** *p* < 0.01, *** *p* < 0.0005, **** *p* < 0.0001.

For the study of an immunomodulatory action of the lentil defensin, several human cell types were chosen. One of the cells representing the first line of defense is pro-inflammatory macrophages (Mφ1). Macrophages are polarized into Mφ1 by microbial products, such as lipopolysaccharide and other ligands of Toll-like receptors (TLRs) invaded into the body. It was found that Lc-def increased the production of inflammatory chemokines MIG (from 82 to 124 pg/mL, *p* < 0.05), MCP-1 (from 169 to 259 pg/mL, *p* < 0.05), and MIP-1β (from 210 to 413 pg/mL, *p* < 0.05) by pro-inflammatory macrophages (Figure 5). Among them, monocyte chemoattractant protein-1 (MCP-1/CCL2) is one of the strongest known chemotactic factors for monocytes and a key chemokine that regulates migration and infiltration of monocytes/macrophages [28].

Monocytes, which are antigen-presenting cells, were shown to produce a broad spectrum of bioactive molecules upon incubation with Lc-def (Figure 5). They are the following growth factors: G-CSF (from below a detectable threshold of <2.57↓ to 22.68 pg/mL, *p* < 0.0001), EGF (2.16 to 11.56 pg/mL, *p* < 0.0001), and PDGF (from below a detectable threshold of <7.38↓ to 321 for AA form; and from 69 to 943 pg/mL for AB/BB form, *p*-values for both forms were <0.05), taking part in tissue reparation and angiogenesis. A number of cytokines was also shown to be elevated: IFNα2 (from 7.77 to 21.43 pg/mL, *p* < 0,05), IL-12(p40) (from 5.21 to 19.56 pg/mL, *p* < 0.05), IL-6 (from 41 to 378 pg/mL, *p* = 0.001), IL-1RA (from 1.4 to 10.83 pg/mL, *p* < 0.01), IL-10 (from 2.21 to 8.4 pg/mL, *p* = 0.01), IL-13 (from below a detectable threshold of <5.00↓ to 15.11 pg/mL, *p* < 0.0001). Interestingly, both pro- (IL-12 and IL-6) and anti-inflammatory cytokines (IL-1RA, IL-10, and IL-13) were secreted by monocytes upon stimulation with Lc-def. The production of several chemokines was also increased: MIG (from 429 to 1406 pg/mL, *p* < 0.01), MIP-1α (from below a detectable threshold of <1.76↓ to 14.98 pg/mL, *p* < 0.05), and MCP-3 (from 82 to 326 pg/mL, *p* < 0.05).

Another cell culture used in the current study was regulatory CD4^+^ FoxP3^+^ T cells (Tregs)—a specialized subpopulation of T cells that acts to suppress immune response, thereby maintaining homeostasis and self-tolerance [29]. It has been shown that Tregs are able to inhibit T cell proliferation and cytokine production and play a critical role in preventing autoimmunity. Tregs are known to have the ability to secrete IL-17. The lentil defensin induced the production of IL-17F by Tregs from below a detectable threshold of <16.96↓ to 51.91 pg/mL, *p* < 0.0001. The production of this pro-inflammatory cytokine was detected only in case of this culture. Lc-def also induced the production of other pro-inflammatory cytokines, chemokines, and growth factors by Tregs: GM-CSF (from 16.28 to 54.75 pg/mL, *p* < 0.0005), IFN-γ (from 9.57 to 38.55 pg/mL, *p* < 0.01), IL-6 (from 192 to 317 pg/mL, *p* < 0.05), TNF-α (from 56.48 to 97.83 pg/mL, *p* < 0.01), MCP-1 (from 195 to 505 pg/mL, *p* < 0.0001), MIP-1α (from 143 to 233 pg/mL, *p* < 0.05), IP-10 (from 30.46 to 114.68 pg/mL, *p* < 0.05), and the only one anti-inflammatory IL-10 (from 13.34 to 42.25 pg/mL, *p* < 0.05) (Figure 5). Thus, in contrast to monocytes that induced both pro- and anti-inflammatory responses upon incubation with Lc-def, Tregs produced mainly pro-inflammatory factors.

We also analyzed secretion profiles of professional antigen presenting cells—dendritic cells (DCs). As mature blood dendritic cells normally represent only ∼0.2% of human PBMC, we applied a commonly used approach to generate mature DCs from blood monocytes. Stimulation with Lc-def did not induce significant changes in cytokine profiles of moDCs in vitro. CD4^+^ T-helper and CD8^+^ T cytotoxic cells represent key effector cells of human immune system. No effects of the lentil defensin on either CD4^+^ T-helper or CD8^+^ T cytotoxic cells were observed. Lc-def also had no effect on the polarized Caco-2 monolayer as a simplified model of the intestinal epithelium (Supporting Materials, Table S2).

## 4. Discussion

In the present work, we investigated the lentil *Lens culinaris* defensin Lc-def discovered by us earlier and studied its antifungal activity against fungi of different *Candida* species, sensitivity to heating and proteolysis, and its effect on human immune cells.

Previously, we showed that the lentil defensin was active against some, but not all, tested phytopathogenic filamentous fungi including *Aspergillus niger*. At the same time, Lc-def did not affect the growth of Gram-positive or Gram-negative bacteria [19,20]. Here, we investigated an ability of Lc-def to inhibit the growth of clinical isolates of *Candida albicans*, *C. krusei*, and *C. glabrata.* An antifungal activity against *Candida* species has been shown previously for some plant defensins. For example, the pea defensin Psd1 at the concentration of 20 μM and PvD1 from the common bean at 18.4 μM caused 100% [13] and 90% [15], respectively, growth inhibition of *C. albicans*. In our case, Lc-def inhibited the growth of all the three tested yeast-like fungi from the *Candida* genus by 80 or 90% at quite high concentration of 50 μM. At the same time, Lc-def did not show any cytotoxic activity towards erythrocytes, as well as such human immune cells as PBMCs and THP-1 cells. 

It is known that one of disadvantages of AMPs is their sensitivity to proteolysis and a rapid degradation by human or pathogenic microorganism proteolytic enzymes. The secreted and membrane-bound proteolytic enzymes are important virulence factors of fungal pathogens. Moreover, a variety of proteases are released upon lysis of microbial cells [4,30]. Here, we investigated for the first time a sensitivity of plant defensins to proteolytic enzymes of *C. albicans* using not only the lentil Lc-def, but also the pea Psd1. The human AMP, cathelicidin LL-37, was used in these experiments for comparison. It was shown earlier that LL-37, possessing a pronounced antifungal activity against *C. albicans*, is effectively degraded by this pathogen [31]. Both plant defensins showed a remarkable stability to proteolysis as compared to LL-37. It is worth mentioning that, despite the absence of the lentil defensin cleavage, its presence in the reaction mixtures with different pH (4.0 and 7.2) decreased the rate of degradation of own proteins of *C. albicans* and α-casein, respectively. This effect was expressed to a lesser extent and was registered only at pH 4.0 in the case of Psd1. At the same time, Lc-def did not affect the degradation rate of LL-37. We hypothesized that different enzymes could be involved in the degradation of α-casein and LL-37 and the presence of Lc-def could affect the work of some of them.

Antifungal drugs are mainly administrated as solutions for intramuscular and intravenous injections, as well as different topical and oral dosage forms. In addition, defensins are present in plant foods consumed fresh or cooked, as in the case of lentil. A high stability of plant defensins has been shown previously. It was revealed, for example, that defensin from the northeast red bean kept antifungal activity after heating and incubation in the solutions of a broad pH range [32]. Using CD spectroscopy, we showed that partial denaturation of the Lc-def structure took place in acidic solution, simulated gastric fluid, and upon defensin heating at 98 ℃. However, the presence of the negatively charged lysolipid LPPG at a high concentration of 1 mM not only increased the α-helix content in the defensin structure, but also prevented its heat denaturation. A high stability of the tobacco NaD1 and the defensin SBI6 from soybean was shown in degradation experiments using pepsin or trypsin [33]. We examined susceptibility of the intact and preheated Lc-def from lentil seeds to proteolytic cleavage mimicking gastrointestinal digestion of the defensin in vitro. In these experiments Lc-def was digested consequently by pepsin and by the mixture of trypsin/α-chymotrypsin. Lc-def was very stable to the cleavage by human digestive enzymes, but preheating led to a partial lentil defensin degradation. This is in a good agreement with data obtained by CD spectroscopy which showed a partial Lc-def denaturation upon heating. Earlier, we showed that disulfide bonds play a critical role in stabilization of the Lc-def structure and the high peptide stability to trypsinolysis. Their reduction leads to a rapid defensin digestion [19]. Therefore, we assumed that heating up to 98 ℃ led to a partial disulfide bonds disruption and increase in the rate of the Lc-def gastrointestinal digestion.

Several cytokines were shown to be essential for immunity to candidiasis [5]. Historically, pro-inflammatory cytokine IL-12 was considered as an essential component of the adaptive response that led to the generation of Th1-type cytokine responses, especially IFN-γ, and protection against disseminated candidiasis [34]. The existence of a positive regulatory loop between pro-inflammatory IL-12 and anti-inflammatory IL-10 was also reported. This may adversely affect the innate antifungal response but is required for optimal co-stimulation of IL-12-dependent CD4^+^Th1 cells [35]. Later, an important role of another pro-inflammatory cytokine, IL-17, and Th17 cells to mucosal candidiasis was confirmed [36]. Here, we reported new data regarding immunomodulatory action of plant defensins on human immune cells using the lentil defensin Lc-def as a case study. In the present work, by using a broad immunology multiplex panel (48 cytokines, chemokines, and growth factors) we demonstrated that the lentil defensin at the concentration of 2 μM exhibited a different immunomodulatory action on various human cell cultures. In particular, Lc-def induced the in vitro production of both IL-12 and IL-10 by primary human monocytes along with other pro-inflammatory cytokines. We also showed that regulatory CD4^+^ FoxP3^+^ T cells (Tregs) secreted increased levels of IL-17 upon stimulation with Lc-def. 

## 5. Conclusions

Taking together all the data obtained, we demonstrated that the lentil defensin Lc-def exhibits an antifungal activity against the clinical isolates of three *Candida* species but has no cytotoxic action on human cells. At the same time, Lc-def shows a high stability to proteolysis by both *Candida albicans* and human digestive enzymes. Moreover, Lc-def affects human immune cells at a low concentration of 2 µM and increases the production of a number of pro- and anti-inflammatory cytokines, including IL-12 and IL-17, essential for immunity to candidiasis. We hypothesized that the lentil Lc-def and plant defensins in general may be effective in suppressing of mucocutaneous candidiasis due to their antifungal activity, high structural stability, and ability to activate a protective immune response. Immunomodulatory effects of the lentil defensin using in vitro and in vivo models of candidiasis would be interesting to evaluate in more detail in prospective studies.

## Figures and Tables

**Table 1:** Secondary structure estimation (%) predicted from far-UV CD spectra.

Sample	Conditions	α-Helix, %	β-Sheet, %	β-Turn, %	Random, %	NRMSD
Lc-def	pH 7.4	15.5	28.5	22.2	33.9	0.03
	pH 2.5	13.9	25.4	24.8	35.8	0.03
Psd1	pH 7.4	14.8	31.8	22.8	30.7	0.03
Lc-def (pH 7.4)	20 °C	15.9	27.7	22.3	34	0.02
	98 °C	13.3	29.6	21.8	35.3	0.08
	20 °C end	9	32.1	22.9	35.9	0.03
Lc-def +LPPG (pH 7.4)	20 °C	23.6	21	22.4	33	0.02
	98 °C	18.5	24.1	22.4	35	0.02
	20 °C end	21.7	23.1	22.2	32.9	0.02
Lc-def +LPPC (pH 7.4)	20 °C	16.4	30.3	22.3	31	0.02
	98 °C	8.1	35.7	22.5	33.7	0.04
	20 °C end	5.8	39.9	21.5	32.8	0.05

## Data Availability

All data generated and analyzed during this study are included in this published article and its Supplementary Materials file.

## Supplementary Materials

The following data are available online at https://www.mdpi.com/article/10.3390/membranes12090855/s1, Figure S1: MALDI mass spectra of the recombinant defensins; Figure S2: (A) Comparison of the amino acid sequences of two legumes defensins—lentil Lc-def and pea Psd1. Identical and similar amino acids are shown in green and blue, respectively. %I, percentage of sequence identity. (B) ELISA with lentil Lc-def and pea Psd1 using rabbit polyclonal anti-Lc-def antibodies. Error bars represent standard deviation between technical replications. (C) SDS-PAGE analysis or (D) immunoblotting with rabbit polyclonal anti-Lc-def antibodies of Lc-def in the absence (1) or present (2) of BME and lysate of *E. coli* cells (3) expressing the fusion protein 8His-Trxl-Lc-def (MW 19kDa); Figure S3: (A) The growth of the clinical isolate of *C. albicans* in the presence of different concentrations of lentil lipid transfer protein Lc-LTP2 (100, 50, 25, 12.5, 6.25, 3.125, and 1.56 μM). Asterisk (red) shows optical density of the control without peptide. (B) Hemolytic assay of different concentrations of Lc-def and melittin as positive control. Error bars represent standard deviation between technical replications; Figure S4: (A) Effect of heating on the sensitivity of Lc-def to digestive enzymes as detected by means of RP-HPLC on Luna C_18_ column (5 μm, 250 × 4.6 mm; Phenomenex) at a flow rate of 0.5 mL/min, using a linear gradient of acetonitrile concentration from 5 to 80% for 60 min in 0.1% trifluoroacetic acid (0 ‘ and 120′ p—pepsin digestion during 10 s and 120 min; 120′ and 24 h p+ch—subsequent digestion by mixture trypsin/α-chymotrypsin during 120 min and 24 h). (B) MALDI mass spectra of the RP-HPLC fractions (from 28, 31, and 33 min) of Lc-def digests; Table S1: List of overlapping primers; Table S2: Absolute levels of 48 cytokines, chemokines, and growth factors assessed by multiplex xMAP technology. Values highlighted in black indicate that the data fall below the low detectable limit, while in blue indicate that the data are in the detectable range; C corresponds to control samples.
