# Supplementary material for: Antifungal Activity, Structural Stability, and Immunomodulatory Effects on Human Immune Cells of Defensin from the Lentil Lens culinaris
Source: Membranes (Basel). 2022 Aug 31;12(9):855. doi: 10.3390/membranes12090855 (PMC9503459; doi:10.3390/membranes12090855)
Supplement: Supplementary file 1 [file membranes-12-00855-s001.zip › membranes-1850596.supp.proof.docx]

Supplementary information for

Antifungal Activity, Structural Stability, and
Immunomodulatory Effects on Human Immune Cells of
Defensin from the Lentil *Lens culinaris*

Ekaterina I. Finkina ^1,^*, Ivan V. Bogdanov ^1^, Anastasia A. Ignatova ^1^, Marina D. Kanushkina ^1^,
Ekaterina A. Egorova ^2^, Alexander D. Voropaev ^2^, Elena A. Stukacheva ^1^ and Tatiana V. Ovchinnikova ^1^

^1^ M.M. Shemyakin & Yu.A. Ovchinnikov Institute of Bioorganic Chemistry, the Russian Academy of Sciences, Miklukho-Maklaya str. 16/10, 117997 Moscow, Russia

^2^ G.N. Gabrichevsky Research Institute for Epidemiology and Microbiology, Admiral Makarov str. 10,
125212 Moscow, Russia

***** Correspondence: finkina@mail.ru; Tel.: +7-495-335-42-00

**Table S1.** List of overlapping primers.

| **№** | **primer sequences (5’-3’)** |
| --- | --- |
| s1 | CCTCGACGCTAACCTGGCCGGATCTATGAAAACTTGTGAACACTTGGCTGATACCTA |
| s2 | CTTGGCTGATACCTACCGTGGAGTTTGTTTCACTAATGCTAGTTGTGACGATCACTGTAAAAA |
| ans3 | CATTTCCAATTATGACACGTGCCTGATATTAGATGTGCTTTATTTTTACAGTGATCG |
| ans4 | GGTGCTCGAGAGAATTCGCGGATCCTTAACAGTTTTGGGTACAGAAGCATTTCCAATTATGACAC |


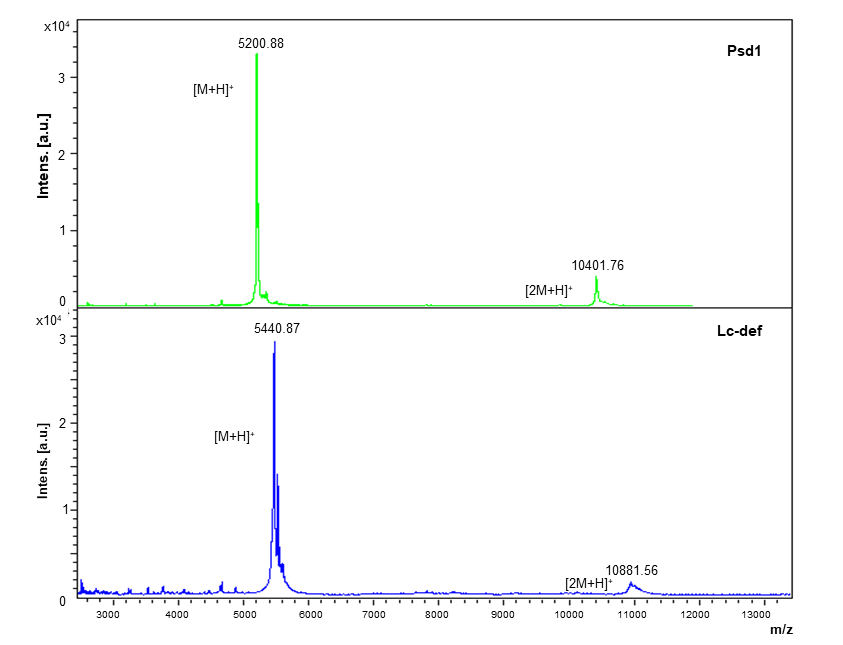


**Figure S1.** MALDI mass spectra of the recombinant defensins.


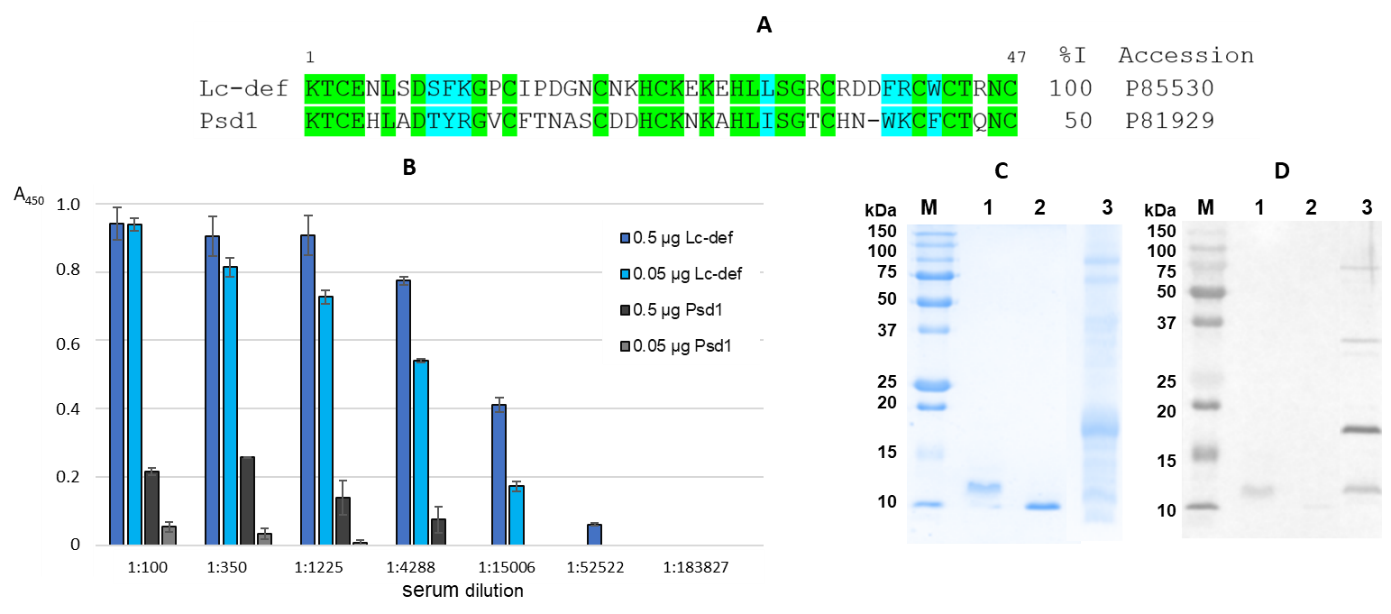


**Figure S2.** (**A**) Comparison of the amino acid sequences of two legumes defensins – lentil Lc-def and pea Psd1. Identical and similar amino acids are shown in green and blue, respectively. %I, percentage of sequence identity. (**B**) ELISA with lentil Lc-def and pea Psd1 using rabbit polyclonal anti-Lc-def antibodies. Error bars represent standard deviation between technical replications. (**C**) SDS-PAGE analysis or (**D**) immunoblotting with rabbit polyclonal anti-Lc-def antibodies of Lc-def in the absence (**1**) or present (**2**) of BME and lysate of *E. coli* cells (**3**) expressing the fusion protein 8His-Trxl-Lc-def (MW 19kDa).


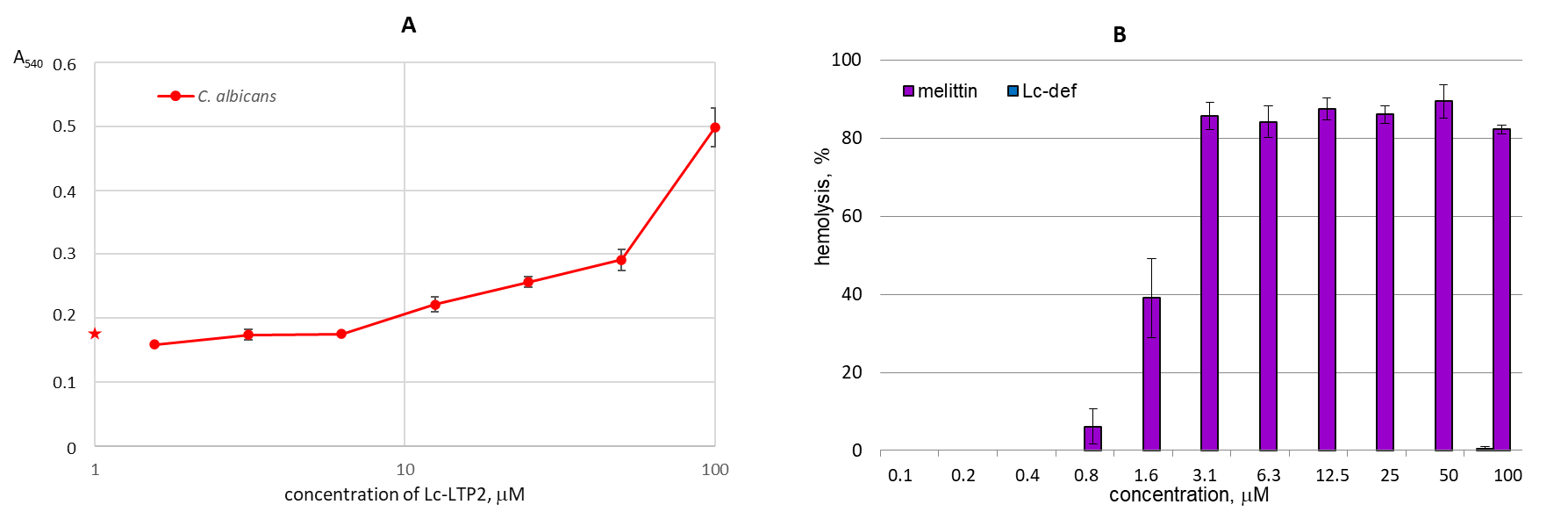


**Figure S3.** (**A**) The growth of the clinical isolate of *C. albicans* in the presence of different concentrations of lentil lipid transfer protein Lc-LTP2 (100, 50, 25, 12.5, 6.25, 3.125 and 1.56 μM). Asterisk (red) shows optical density of the control without peptide. (**B**) Hemolytic assay of different concentrations of Lc-def and melittin as positive control. Error bars represent standard deviation between technical replications.


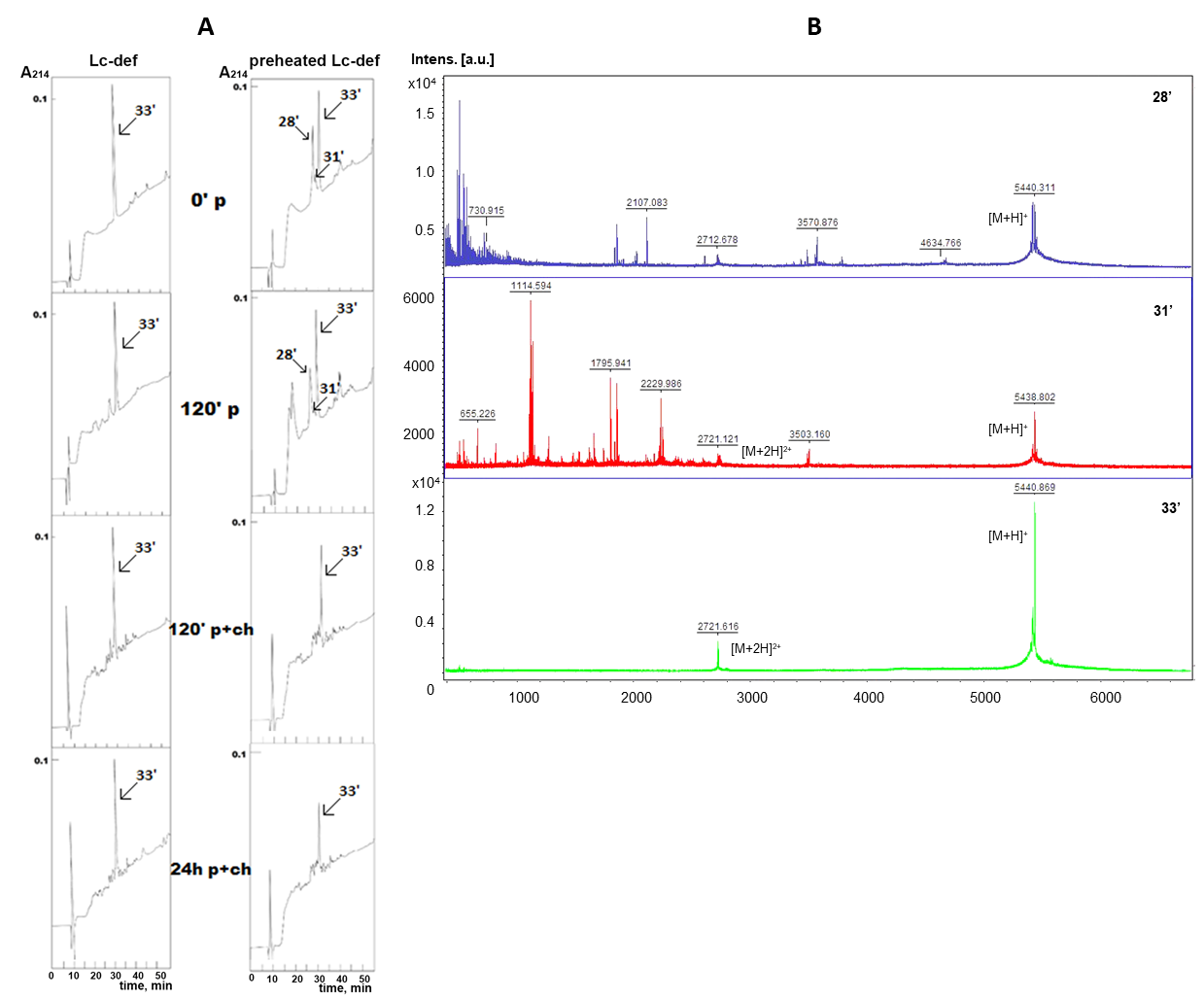


**Figure S4.** (**A**) Effect of heating on the sensitivity of Lc-def to digestive enzymes as detected by means of RP-HPLC on Luna C_18_ column (5 μm, 250 × 4.6 mm; Phenomenex) at a flow rate of 0.5 mL/min, using a linear gradient of acetonitrile concentration from 5 to 80% for 60 min in 0.1% trifluoroacetic acid (0 ‘ and 120’ p— pepsin digestion during 10 s and 120 min; 120’ and 24 h p+ch —subsequent digestion by mixture trypsin/α-chymotrypsin during 120 min and 24 h). (**B**) MALDI mass spectra of the RP-HPLC fractions (from 28, 31 and 33 min) of Lc-def digests.
